# Indian community health insurance schemes provide partial protection against catastrophic health expenditure

**DOI:** 10.1186/1472-6963-7-43

**Published:** 2007-03-15

**Authors:** Narayanan Devadasan, Bart Criel, Wim Van Damme, Kent Ranson, Patrick Van der Stuyft

**Affiliations:** 1 Achutha Menon Centre for Health Science Studies, SCTIMST, Thiruvananthapuram, Kerala, India; 2 Department of Public Health, Institute of Tropical Medicine, Antwerp, Belgium; 3 Honorary Lecturer, Health Policy Unit, London School of Hygiene and Tropical Medicine, London, UK

## Abstract

**Background:**

More than 72% of health expenditure in India is financed by individual households at the time of illness through out-of-pocket payments. This is a highly regressive way of financing health care and sometimes leads to impoverishment. Health insurance is recommended as a measure to protect households from such catastrophic health expenditure (CHE). We studied two Indian community health insurance (CHI) schemes, ACCORD and SEWA, to determine whether insured households are protected from CHE.

**Methods:**

ACCORD provides health insurance cover for the indigenous population, living in Gudalur, Tamil Nadu. SEWA provides insurance cover for self employed women in the state of Gujarat. Both cover hospitalisation expenses, but only upto a maximum limit of US$23 and US$45, respectively.

We reviewed the insurance claims registers in both schemes and identified patients who were hospitalised during the period 01/04/2003 to 31/03/2004. Details of their diagnoses, places and costs of treatment and self-reported annual incomes were obtained. There is no single definition of CHE and none of these have been validated. For this research, we used the following definition; "*annual hospital expenditure greater than 10% of annual income*," to identify those who experienced CHE.

**Results:**

There were a total of 683 and 3152 hospital admissions at ACCORD and SEWA, respectively. In the absence of the CHI scheme, all of the patients at ACCORD and SEWA would have had to pay OOP for their hospitalisation. With the CHI scheme, 67% and 34% of patients did not have to make any out-of-pocket (OOP) payment for their hospital expenses at ACCORD and SEWA, respectively. Both CHI schemes halved the number of households that would have experienced CHE by covering hospital costs. However, despite this, 4% and 23% of households with admissions still experienced CHE at ACCORD and SEWA, respectively. This was related to the following conditions: low annual income, benefit packages with low maximum limits, exclusion of some conditions from the benefit package, and use of the private sector for admissions.

**Conclusion:**

CHI appears to be effective at halving the incidence of CHE among hospitalised patients. This protection could be further enhanced by improving the design of the CHI schemes, especially by increasing the upper limits of benefit packages, minimising exclusions and controlling costs.

## Background

Out-of-pocket payments by individual households are the main source of health care financing in India. National health accounts show that 72% of all health expenditure is made by individual households [1] which is one of the highest proportions in the world [2]. Estimates from consumer expenditure surveys show that an Indian household spends an average of 5% of its total expenditure on health care [3].

Contrary to most other consumption expenses, medical expenditure is largely unpredictable both in timing and quantity. Households, especially in low income countries, cope either by divesting their savings, borrowing, mortgaging or selling assets, or by forgoing treatment [4-10]. Many households are impoverished because of medical expenses. A study covering 5 districts in Rajasthan, India showed that medical expense was one of the three main factors pushing people into poverty [11]. A nationally representative sample survey indicated that an additional 37 million Indians (3.7% of total population) were impoverished in the year 1999 because of health care costs; increasing poverty head counts by 12% [12]. Still other studies show that 17 to 34% of hospitalised Indian patients are impoverished because of medical costs [13]. Such health care expenditures that have an adverse impact on the household are usually termed "*catastrophic health expenditures (CHE)*".

Health insurance is put forward as a measure to protect against CHE [4,9]. While this is theoretically plausible, there is little empirical evidence from low-income countries to support this hypothesis [14]. While Ranson [15] has demonstrated a reduction in CHE in one community health insurance (CHI) scheme, in this article we explore whether the same effect is observed in another CHI scheme in India. We studied two Indian CHI schemes to investigate whether they reduced the incidence and intensity of CHE. A second objective was to identify some of the determinants of CHE in the Indian context. Our research hypotheses were:

1. CHI schemes protect households from catastrophic health expenditure

2. The household income, the depth of the benefit package and the cost of health care determine the incidence and intensity of CHE.

Finally, based on our findings, we explore how these schemes' protection against CHE can be enhanced.

This is a comparative study, measuring the effect of two CHI schemes on CHE. For this study, we purposively selected two schemes that had different design features – ACCORD and SEWA (as documented below).

## Context

ACCORD, a non-governmental organisation (NGO) in Tamil Nadu, south India, works for the overall development of the indigenous people of the Gudalur sub-district. This population also called 'adivasis' have traditionally been hunters and food gatherers. However, with progressive deforestation over the past few decades, most of them have shifted to wage labour. As per the 2001 census, there were 215,269 inhabitants in Gudalur, of which 14,149 were adivasis [16]. ACCORD collaborates with a community based organisation, the Adivasi Munnetra Sangam (AMS), to fight for adivasi rights. In addition, ACCORD also provides health, education and agricultural services for the adivasis.

ACCORD's health programme (ASHWINI) is a three tier health system, with village health workers, health centres and a 20 bed hospital. Other than the ASHWINI hospital, there are four other NGO hospitals with a total of 75 beds, three government hospital (160 beds) and one private hospital (10 beds) in Gudalur sub district. There are only four specialists in Gudalur, two in the ASHWINI hospital and two in the government hospitals.

**Figure 1:**
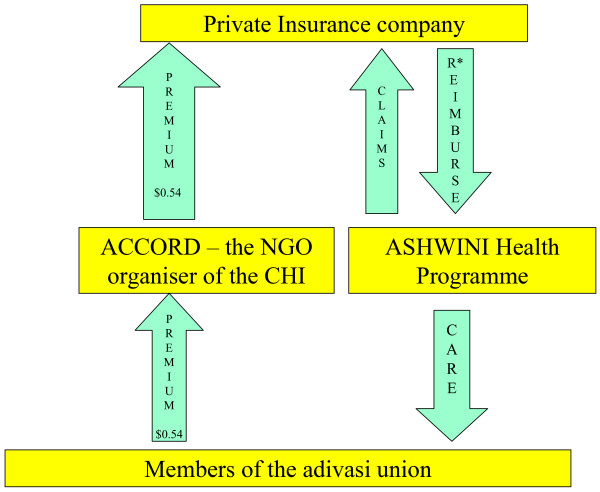
The ACCORD community health insurance scheme in 2003.

Part of the ACCORD health service is financed by a CHI scheme initiated in 1992[17]. All AMS members and their households are eligible to join the ACCORD CHI scheme (Figure 1). In 2003, each AMS member paid a premium of Rs 25 (US$0.54) per person per year during a definite collection period. This premium was collected by ACCORD and ASHWINI field staff and AMS leaders. Primary care was provided free to all adivasis, irrespective of their insurance status, by health staff at village and health centre levels. Insured members, if hospitalised in the ASHWINI hospital, were entitled to hospital care up to a maximum limit of Rs 1,000 (US$23). Non-insured AMS members had to meet the costs of medicines (between US$2 and US$5), while non-adivasi patients had to pay the entire hospital bill (between US$15 and US$20). This entire CHI scheme was jointly managed by ACCORD and ASHWINI staff and AMS leaders. In turn, ASHWINI reinsured the adivasis with a private health insurance company.

**Figure 2:**
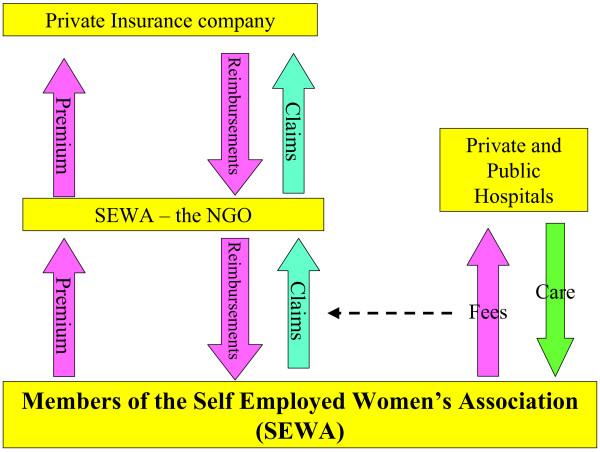
The SEWA community health insurance scheme in 2003.

The Self Employed Women's Association (SEWA) is a union of women employed in the informal sector. While its main area of operation is Gujarat, it also has presence in other states of India. Among its diverse activities, it provides an integrated insurance package – including life, asset and medical insurance – on a voluntary basis, for its members and their husbands [18]. In 2003, for an annual premium equivalent to US $3.20, a couple was insured for hospital services up to a maximum of US $45 (Figure 2). The patients could access care in either the public or the private sector. Patients had to pay the hospital bills and were reimbursed later by SEWA after producing the necessary documents.

In Gujarat, like in most Indian states, there is a network of public and private providers, operating independent of each other. While the government hospitals are based on population norms: a community health centre with 30 beds for 100,000 population, a sub-district hospital with 100 beds for 500,000 population and a district hospital with 200 beds for a million population, the private sector is more diverse and disparate. Most of the private sector hospitals are located at the district level have just 6 – 30 beds and limited facilities [19].

Both CHI schemes, in principle target the poor and vulnerable sections of Indian society. While both are organised by NGOs and insure against hospital expenditure, there were some differences. First, at the ACCORD CHI, all members of the AMS and their family were eligible to enrol. At SEWA, only the woman member and her spouse were eligible to enrol in 2003. Secondly, the ACCORD CHI scheme was a cashless one, so the patient does not have to pay anything as long as the hospital bill was less than US$23. At SEWA, the patient had to pay the bills to the provider and was reimbursed later up to a maximum of US$45. Furthermore, the ACCORD CHI scheme recognised only one provider, the ASHWINI hospital, which is a not-for-profit institution. Patients enrolled in the SEWA scheme sought care at both private and government hospitals. Lastly, while the ACCORD CHI scheme had minimal exclusions, SEWA usually excluded pre-existing illnesses from reimbursement, especially during the member's first year as a policy-holder. Both schemes were supported by external donors to a certain extent.

## Methods

At both ACCORD and SEWA, we reviewed the insurance claims registers and identified all the scheme members who were hospitalized between the period April 2003 to March 2004, and who registered this hospitalization with the scheme. For these patients, we collected details on the age and sex of the patient, diagnosis, total bill amount and amount paid out-of-pocket by the patient. At SEWA, we also documented the amount reimbursed subsequently and the annual household income as reported by the patient from the insurance claim register. At ACCORD, we used the median annual household expenditure obtained from an independent survey (personal communication 2004) to estimate the annual income of the households with patients.

There is still a lot of debate on the definition of CHE. Most authors agree that health expenditure is catastrophic if it forces households to significantly lower their standard of living now or in the future. While some have defined CHE if the total health expenditure is more than 10% of annual income [5,12,15], others have defined it if the total health expenditure exceeds 40% of disposable income [4]. For this study we have opted for a modified version of Pradhan's definition of CHE i.e. "*if a household expenditure for hospitalisation exceeded 10% of the total annual household income*". We used this definition because, according to literature, 10% of total expenditure is considered as an approximate threshold at which the poor household is forced to sacrifice other basic needs, sell productive assets or incur debt [20]. Furthermore, this is a simple calculation and does not require details of other expenditure like the expenditure on food.

Using this definition, we calculated the incidence of out-of-pocket (OOP) payments and CHE. To calculate the OOP before insurance, we used the gross hospitalisation expenditure at both ACCORD and SEWA. We then expressed this expenditure as a proportion of the reported annual income. If this was more than 10%, then that household was considered to have experienced CHE. OOP payment after insurance was calculated by subtracting the total hospital expenditure from the amount reimbursed. In the case of ACCORD it was the amount that the patient had to pay at discharge (if the hospital bill exceeded the upper limit). CHE was then calculated by computing this OOP payment (post insurance) as a proportion of the reported annual income. If this was more than 10%, then that household was considered to have experienced CHE. The difference gives us an idea of the effect of the CHI scheme on CHE among these poor households. We compared the medians and used the 95% confidence intervals to analyse any differences between the two schemes. To compare the incidence of CHE by specific characteristics, we used the risk ratio and the 95% confidence interval. As we did not have the individual household income at ACCORD, we limited the analysis of determinants to the SEWA scheme.

Ethical clearance for this study was provided by the ethical board of the Sri Chitra Tirunal Institute of Medical Sciences and Technology, Trivandrum, Kerala, India.

## Results

In April 2003, a total of 4,268 individuals out of 12,226 AMS members (35%) enrolled in the ACCORD CHI scheme. In the same period, at SEWA, 101,809 women and men out of about 500,000 SEWA members (~20%) enrolled in the CHI scheme (Table 1). 571 individuals from 476 households were admitted a total of 683 times at ACCORD, while at SEWA the corresponding figures were 3080 individuals from 2989 households leading to 3152 admissions. However, we analysed data from only 2974 households as the rest did not have information about hospitalisation expenses or household incomes. The admission rates at ACCORD and SEWA were 134 and 37 per 1000 insured per year, respectively. Females accounted for 59% and 75% of the admissions at ACCORD and SEWA and the median age of the patients was 21 and 36 years, respectively. The median household income (Q1, Q3) at ACCORD was US$630 (518, 813) while at SEWA it was US$545 (273, 818).

**Table 1:** Characteristics of hospitalised patients in the two CHI schemes in India (01/04/2003 to 31/03/2004)

	**ACCORD**	**SEWA**
Number of families insured (individuals)	1,028 (4,268)	83,531 (101,809)
Number of admissions	683	3152
Number of families with admissions	476	2989
Admission rates per 1000 individuals	134	37
Number of female admissions (%)	401 (59%)	2370 (75%)
Median age of patient (Q1, Q3) in years	21 (6, 32)	36 (30, 44) *
Median annual income (Q1, Q3) in US$	630 (518, 813)	545 (273, 818)

* Age of 24 patients were not available in SEWA

While all of the admissions at ACCORD were in the designated not-for-profit institution, the ASHWINI hospital; at SEWA the majority of admissions were in private-for-profit institutions (Table 2). The median hospital bill at ACCORD was US$12, while at SEWA it was US$46. However, because of a combination of low upper limits and exclusions, some of the patients did not benefit from the insurance scheme. This occurred more often in SEWA where 19% of claims were not reimbursed either because their illness was in the list of exclusions or they had crossed the upper limit in an earlier admission. At ACCORD, 74% of all claims were fully covered, while at SEWA only 38% of all claims were fully reimbursed. The rest of the patients were only covered up to the upper limits. This resulted in a reimbursement amount which was significantly lower than the claimed amount at SEWA. This was further compounded by the fact that at SEWA the patients had to mobilise financial resources during the time of illness and were reimbursed later. The median time to reimbursement was six weeks.

**Table 2:** Details of claims in two CHI schemes in India (01/04/2003 to 31/03/2004)

	**ACCORD**	**SEWA**
**Type of provider**		
Total claims	683	3152
in government hospitals	**-**	238 (8%)
in NGO hospitals	683 (100%)	204 (6%)
in private-for-profit hospitals	**-**	2710 (86%)
**Details of claims made**		
Median claim amount (95% CI) in US$	11.8 (10.9, 12.7)	46.4 (45.2, 47.5)
Median claim honoured (95% CI)^@ ^in US$	10.4 (9.5, 11.2)	12.9 (12.7, 13.1)
Number of claims that were honoured (%)	643 (94%)	2543 (81%)
Number of claims that were fully honoured (%)	507 (79%)	1206 (47%)
Number of claims that were partially honoured (%)	136 (21%)	1337 (53%)
Median delay (Q1, Q3) between discharge and reimbursement in days	-	49 (23, 81)
**Type of illnesses**		
Acute illnesses	401 (59%)*	2346 (74%)
Pre-existing illnesses	280 (41%)	806 (26%)

^@ ^The term "honoured" has different implications in the two schemes. At SEWA it means "*reimbursement" *while at ACCORD, it implies the amount paid through direct third party payment.

* Details of 2 episodes not available.

In the absence of health insurance, households at both ACCORD and SEWA would have paid out-of-pocket to meet their hospital bills. The median OOP payments would have been US$18 and US$48, respectively (Table 3). However, thanks to the CHI scheme, 67% of insured households at ACCORD and 34% of insured households at SEWA were protected from making OOP payments. The magnitude of OOP payments also reduced significantly.

**Table 3:** Out of pocket payment (OOP) and catastrophic health expenditure (CHE) among households* insured in the two Indian CHIs (01/04/2003 to 31/03/2004)

	**ACCORD CHI scheme**	**SEWA CHI scheme**
**Number of households that would have paid OOP in the absence of insurance**	476	2974
**Number of households that paid OOP after insurance (%)**	159 (33%)	1953 (66%)
**Median OOP payment (95% CI) by households in the absence of insurance (US$)**	18 (16, 19) (n = 476)	48 (46, 49) (n = 2974)
**Median OOP payment (95% CI) by households after insurance (in US$).**	13 (10, 17) (n = 159)	28 (26, 30) (n = 1953)

**Number of households that would have experienced CHE in the absence of insurance (% with 95% CI)**	39 8.2% (5.8,11.0)	1461 49% (47, 51)
**Number of households that experienced CHE after insurance (% with 95% CI)**	17 3.5% (2.1, 5.6)	694 23% (22, 25)

**Median (95% CI) proportion of annual income that would have been spent on hospitalisation by households experiencing CHE before insurance**	14% (12, 16) (n = 39)	14 % (13, 14) (n = 1461)
**Median (95% CI) of proportion of annual income spent on hospitalisation by households experiencing CHE after insurance**	9% (7, 11) (n = 39)	9% (8, 10) (n = 1461)

* Only those households where data for both hospitalisation expenses and annual income were available were included for these calculations

95% CI = 95% confidence interval

**Figure 3:**
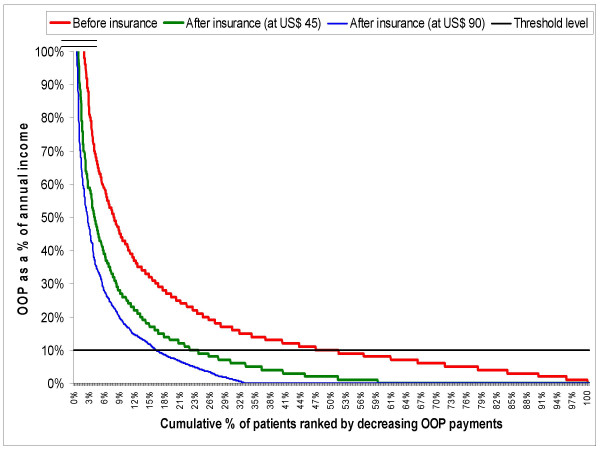
Incidence of catastrophic health expenditure among insured patients in SEWA, India (2003–2004)

Of all the insured households with admissions at ACCORD, 8% would have experienced catastrophic health expenditure (CHE) in the absence of an insurance scheme. However, because of the CHI scheme, this proportion was reduced to 3.5%. At SEWA, 49% of the households would have been catastrophically affected by the admission costs if they were not insured. The CHI scheme at SEWA has been successful in reducing the incidence of CHE in insured households to 23%. Not only has the incidence of CHE been halved in both the schemes, the magnitude of CHE has also been significantly reduced in both schemes. This is graphically represented in Figure 3, where one can see the shift in both the incidence and intensity after insurance by the SEWA CHI scheme. A sensitivity analysis at SEWA indicated that an increase in the upper limit of reimbursement from US$ 45 to US$ 90 would reduce the incidence of CHE after insurance from 23% to 16%.

There are many causes for the phenomenon of CHE. One important factor is high medical bills. The medical bills in these two schemes were very different, nearly four times higher at SEWA. This difference was further exaggerated when one disaggregates the data by providers. At SEWA, the median bill with a private provider was US$48, while it was only US$28 with a government provider. One possibility for this difference in price between the two schemes could be different case mixes. However, even though these schemes are more than 1000 km apart and the populations are different, we note that the case mix is relatively uniform in both the schemes; the cases are a mixture of communicable and non-communicable conditions (Table 4). There were more fever cases at SEWA because of the presence of malaria. At SEWA, pregnancy related conditions were low because this was excluded from the benefit package; while at ACCORD; ocular conditions were always referred to a neighbouring ophthalmic hospital that provided free services for the adivasis. What is striking is that the charges for similar conditions were systematically and significantly more expensive at SEWA. This is most probably because of the reliance on private providers at SEWA. We see from Table 5 that the probability of CHE is high (24%) if the patient has visited a private provider and is nearly two times more than a patient who had visited a government provider.

**Table 4:** Reasons for hospitalisation and associated costs per episode from two CHI schemes in India, (01/04/03 to 31/03/04).

**Top ten reasons for hospitalisation**	**ACCORD**		**SEWA**	
	Number of admissions* (% of total; 95% CI)	**Median claim (US$)** **(95% CI)**	**Number of admissions** **(% of total; 95% CI)**	**Median claim (US$)** **(95% CI)**

Respiratory tract infections incl. TB	**125 **(18%; 15, 21)	**11 **(9, 12)	**306 **(10%; 9, 11)	**46 **(43, 49)
Pregnancy related conditions	**104 **(15%; 12, 18)	**22 **(18, 25)	**31 **(1%; 0.7, 1.4)	**39 **(27, 51)
Diarrhoeal diseases and dysentery	**84 **(12%; 10, 15)	**6 **(5, 7)	**331 **(11%; 9, 11)	**47 **(45, 49)
Fevers	**61 **(9%; 7, 11)	**11 **(9, 12)	**745 **(24%; 22, 25)	**43 **(41, 45)
Non-communicable diseases	**57 **(8%; 6, 10)	**12 **(8, 15)	**264 **(8%; 7, 9)	**50 **(44, 56)
Diseases of the urinary system	**39 **(6%; 4, 8)	**14 **(10, 18)	**142 **(5%; 4, 5)	**48 **(43, 53)
Acute Abdomen (including laparotomies)	**36 **(5%; 4, 7)	**11 **(7, 15)	**174 **(6%; 5, 6)	**52 **(44, 61)
Injuries	**31 **(5%; 3, 6)	**15 **(8, 22)	**405 **(13%; 12, 14)	**36 **(33, 39)
Diseases of the reproductive system (including Hysterectomies)	**32 **(5%; 3, 6)	**22 **(17, 26)	**194 **(6%; 5, 7)	**77 **(67, 87)
Ocular conditions	**0 **(0%)	**0**	**113 **(4%; 3, 4)	**47 **(39, 55)
Others	**112 **(16%; 14, 19)	**11 **(9, 13)	**447 **(14%; 13, 15)	**49 **(45, 53)
All episodes	**681 **(100%)	**12 **(11, 13)	**3152 **(100%)	**46 **(45, 47)

* Details of 2 episodes not available.

Our results also indicate that at SEWA low annual household income predisposes a household towards CHE (Table 5). The SEWA CHI scheme reduced the incidence of CHE by more than 50% for all but the richest quartile. Even then, the probability of the lowest quartile experiencing CHE per episode is six times higher than for the richest quartile after insurance. While there was a reduction in the incidence of CHE by more than two times for most disease conditions, patients with diseases of the reproductive system, patients with acute abdominal pain (including acute surgical illnesses) and patients with non-communicable diseases still had a high probability of CHE. Naturally, those whose claims have been rejected have a much higher probability of CHE, the risk at SEWA being three times higher.

**Table 5:** Incidence of catastrophic health expenditure per episode at SEWA by specific characteristics

	**Number of cases (%)**	**Number of cases with CHE (%) before insurance**	**Number of cases with CHE (%) after insurance**	**Probability of experiencing CHE before insurance (95% CI)**	**Probability of experiencing CHE after insurance (95% CI)**
**Provider**					
Private	2711 (86%)	1339 (89%)	640 (90%)	49% (47, 51)	24% (22, 25)
NGO	204 (6%)	74 (5%)	41 (6%)	36% (30, 43)	20% (15, 26)
Government	236 (8%)	84 (6%)	30 (4%)	35% (29, 42)	13% (9, 18)

**Annual household income**					
Q1	928 (31%)	845 (56%)	396 (57%)	91% (89, 93)	43% (39, 46)
Q2	561 (19%)	346 (23%)	145 (21%)	62% (57, 66)	26% (22, 30)
Q3	869 (29%)	234 (16%)	112 (16%)	27% (24, 30)	13% (11, 15)
Q4	616 (21%)	71 (5%)	41 (6%)	11% (9, 14)	7% (5, 9)

**Diagnosis**					
Fevers	745 (24%)	365 (25%)	126 (18%)	49% (45, 53)	17% (14, 20)
Injuries	405 (13%)	142 (10%)	70 (10%)	35% (30, 40)	17% (14, 21)
Diarrhoeal diseases	331 (11%)	123 (8%)	35 (5%)	37% (32, 42)	11% (7, 14)
Respiratory tract infections	306 (10%)	157 (10%)	66 (9%)	51% (45, 57)	22% (17, 27)
Non-communicable diseases	264 (8%)	139 (9%)	78 (11%)	53% (46, 59)	30% (24, 35)
Acute abdominal conditions (incl. laparotomies)	174 (6%)	100 (7%)	62 (9%)	57% (50, 65)	36% (29, 43)
Diseases of the reproductive system (incl. hysterectomies)	194 (6%)	117 (8%)	87 (12%)	60% (53, 67)	45% (38, 52)
Diseases of the urinary system	142 (5%)	74 (5%)	31 (4%)	52% (44, 61)	22% (15, 30)
Ocular conditions	113 (4%)	44 (3%)	20 (3%)	39% (30, 49)	18% (11, 26)

**Exclusions**					
Claim rejected	608 (19%)	306 (20%)	306 (43%)	50% (46, 54)	50% (46, 54)
Claim accepted	2544 (81%)	1191 (80%)	405 (57%)	47% (45, 49)	16% (14, 17)

95% CI = 95% confidence interval

## Discussion

Protecting households against catastrophic health expenditure is a health policy goal. There is documented evidence to show that health expenditure can impoverish households[4]; make them forego further treatment [5], sell assets [6]; remove children from school [10]; substitute labour [9]; diversify income [21] and even lead to suicide [22]. In India, the two CHI schemes studied were able to halve the number of households that would have faced CHE. The magnitude of OOP payments was significantly reduced in both the schemes. This is a substantial achievement and indicates that these schemes fulfil a significant insurance function [23].

However, this protection is only partial and some households still experience CHE, especially the poorest ones. The incidence of CHE was nearly six times higher among the poorest quartile than among the richest quartile. This is understandable as the poor have low incomes and so any health expenditure can easily exceed 10% of their incomes. This is more so in a scheme like SEWA, where bills are reimbursed after a month or more. Patients have to mobilise resources to pay the bills. Sometimes this is in the form of short term loans from moneylenders who charge high interest rates. However, it is positive to note that the poorer households also benefited the most from the health insurance mechanism. The risk of CHE reduced by more than 50% after insurance in the poorest two quartiles, while in the richest two quartiles, it was less than 50%. This indicates that such schemes are still pro-poor in their financial protection function.

One of the limitations of our study is that it is facility based. For estimating CHE, one should consider all health expenditure during a full year. However, here we have assessed the household expenses only for hospitalisation; therefore, the real incidence and intensity of CHE is underestimated in our study. However, the high bills associated with a hospitalisation generally has a much larger impact than expenses for ambulatory or preventive care that are lower in magnitude and spread over time. So, calculating CHE based on hospitalisation expenses gives us a fair idea of the economic shock experienced by a household. We do however recognise that that for some households, even expenditure on medicines can be catastrophic.

At SEWA, patients who had crossed the upper limit in the first admission may not have submitted a second claim, knowing that they would not be reimbursed. This further compounds our underestimate. As we did not have information on the individual household incomes of the patients at ACCORD, we had to use survey data as a proxy for calculating the CHE. So our calculation of CHE in ACCORD is actually the proportion of the median household income. This naturally would result in an underestimation of the incidence of CHE in the poor, while there would be an overestimation among the better off. This disparity would depend a lot on the variation of the income within the community. But, as most adivasi households at ACCORD are relatively homogenous in their poverty, we feel that this factor should not affect the results significantly.

As mentioned earlier, there is considerable debate on the definition of CHE. While some authors put the threshold at 10% of the annual income, others use the indicator based on disposable income. Which is more valid? Is it relevant to have an uniform threshold, irrespective of the economic status? It may be presumed that in a poor household, even a small proportion spent on health care may be catastrophic. On the other hand a better off household may be able to absorb the shock of a higher proportion of its income spent on health care. Hence to put uniform thresholds for all households is somewhat arbitrary and misleading. Similarly can this indicator be equally applied to subsistence farmers? These households have very little cash transaction and hence even a small expenditure may be catastrophic, especially if it means that they have to sell their future food supply. These and other questions beg answers, which need detailed validation studies. It was not our purpose to validate any of these indicators, but to show that even in the presence of insurance, households do still continue to experience CHE.

The fact that health expenditure continues to lead to CHE despite insurance coverage is a concern and is a consequence of various factors. One reason is the low maximum limit in both schemes. One way of increasing the protective effect of CHI schemes would be to expand the maximum limit of the benefit package to cover common surgical and medical conditions. However, any increase in the benefit package would be associated with a rise in the premium. This may adversely affect enrolment. One possible solution is for government or donors to subsidise the premiums, especially for the poorer households. This would allow the poor to enrol in the scheme as well as protect them from CHE.

It was surprising to see that though SEWA has a higher upper limit, more patients pay OOP and more households experience CHE. One important reason could be the higher bills at SEWA. Another measure to reduce the risk of CHE would thus be to reduce the costs of health care. From our data we note that the prices of hospital services for similar conditions were systematically higher at SEWA compared to ACCORD. This could be because of the predominant use of the private sector in SEWA. In India, CHI schemes may be forced to use the private health services if they want to provide their community with choices; but in this case, they would need to introduce certain cost containment measures. One measure could be to pay providers on a case basis, rather than on a fee-for-service basis [24]. This in itself would reduce the danger of unnecessary interventions.

This tendency for health insurance to increase the total health care expenditure of households is described by Bogg et al in their study in China [25]. They show that in two neighbouring districts, one with a CHI scheme covering the population and another with user fees; the former district had higher total health expenditure over time, mainly for curative care and with over prescription of medicines being the rule of the day. A second measure to reduce costs is to insist on the use of generics and standard treatment protocols for common ailments. If this could be combined with other technical tools like medical audits and appropriate evaluation protocols [26], then the quality of the care provided could also be considerably enhanced. Thus, CHI schemes could be used not only to provide financial protection for households, but could also improve the quality of the services by acting as a "strategic purchaser" of health care services [27]. The latter would be useful in a situation where the quality of care in the private sector is questionable [28].

Exclusions in a health insurance scheme are detrimental for various reasons. From the patient's perspective, it adds to the uncertainty at the time of care. From a public health perspective, it does not provide protection for those patients who are the most vulnerable i.e. patients with chronic illness and who require regular medications [29]. Finally, it is in conflict with the principles of social health insurance. This study shows that even poor populations have a significant prevalence of non-communicable diseases and that exclusion of these conditions can have an economic impact. Patients whose claims have been rejected because of exclusions have a higher probability of CHE. This has policy implications and designers of CHI schemes need to ensure that exclusions are minimised and that people are covered for a comprehensive range of illnesses.

## Conclusion

CHE is a major cause of impoverishment and patients need to be protected from it [29]. Some of the documented determinants of CHE are poverty, household size, high medical costs, incidence of illness, payment mechanisms, low benefit packages and presence of 'smokers or drinkers' in the household [4,30-33]. We show here that the incidence of CHE is also related to the type of provider; private-for-profit providers considerably increase the probability of CHE. We have documented some of the illnesses that can lead to CHE, namely surgical ailments and admissions for non-communicable diseases.

Indian CHI schemes are able to protect their members against CHE, but only to a limited level. However, this protection can be further enhanced if some design changes are incorporated. To begin with, the upper limit of the benefit package needs to be raised. To keep the premiums affordable, donors or the government would need to directly subsidise the premium, especially for the poorer sections of society. Exclusions need to be minimised to protect vulnerable populations. And finally, scheme managers need to negotiate costs with providers from the start [34] to ensure that costs are contained. Such measures could considerably reduce the incidence and magnitude of CHE and protect households from iatrogenic poverty [29].

NB: Subsequent to our study, SEWA has made significant changes in its design, including expansion of eligibility to include all members of the family and piloting of a third party payment mechanism. ACCORD has increased its upper limit from US$ 23 to US$ 69.

## Abbreviations

**ACCORD **Action for Community Organisation, Rehabilitation and Development

**AMS **Adivasi Munnetra Sangam

**ASHWINI **Association for Health Welfare in the Nilgiris

**SEWA **Self Employed Women's Association

**NGO **Non-governmental organisation

**CHE **Catastrophic health expenditure

**CHI **Community health insurance

**OOP **Out-of-pocket

**SCTIMST **Sree Chitra Tirunal Institute for Medical Sciences and Technology

## Competing interests

This is certify that Dr. N. Devadasan, was the founder of the ACCORD CHI. He is however, not directly involved with the project anymore and is currently a visiting faculty at SCTIMST and pursuing a PhD with the ITM, Antwerp.

Dr MK Ranson was involved with SEWA while pursuing his PhD and post doctoral research.

Dr. Wim van Damme, Dr Bart Criel and Dr Patrick Van der Stuyft have not been associated either with ACCORD or with SEWA.

The authors declare no financial competing interest.

## Authors' contributions

ND conceptualised the study and participated in it by collecting the data at ACCORD, analysing and interpreting the data and drafting the manuscript. WVD conceptualised the study, assisted in interpretation of the data and revised the manuscript critically. KR participated in the study by collecting the data at SEWA and revised the manuscript critically. BC assisted in interpretation of the data and revised the manuscript critically for substantial intellectual content. PVDS conceptualised the study and assisted in interpretation of the data and revised the manuscript critically for substantial intellectual content.

All the authors confirm that they had access to all the data, have read and approved the final manuscript.

## Pre-publication history

The pre-publication history for this paper can be accessed here:



## References

[B1] Ministry of Health & Family Welfare (2006). National Health Accounts, India.

[B2] WHO (2006). The world health report 2006 – working together for health.

[B3] Garg CC, Karan AK (2005). Health and Millennium Development Goal 1: Reducing out-of-pocket expenditures to reduce income poverty – Evidence from India.

[B4] Ke X, Evans DB, Kawabata K, Zeramdini R, Klavus J, Murray CJ (2003). Household catastrophic health expenditure: a multi-country analysis. Lancet.

[B5] Pradhan M, Prescott N (2002). Social risk management options for medical care in Indonesia. Health Economics.

[B6] Van Damme W, Van Leemput L, Por I, Hardemann W, Meessen B (2004). Out-of-pocket health expenditure and debt in poor households: evidence from Cambodia. Trop Med Int Health.

[B7] Gertler P, Levine DI, Moretti E (2003). Do Microfinance Programs Help Families Insure Consumption Against Illness?.

[B8] (2002). Incidence of non-fatal health outcomes and debt in urban India.

[B9] Sauerborn R, Adams A, Hien M (1996). Household strategies to cope with the economic costs of illness. Soc Sci Med.

[B10] Van Damme W, Meessen B, Por I, Kober K (2003). Catastrophic Health Expenditure [Letter]. Lancet.

[B11] Krishna A (2004). Escaping poverty and becoming poor: who gains, who loses and why?. World Development.

[B12] van Doorslaer E, O'Donnell O, Rannan-Eliya RP, Samanathan A, Adhikari SR, Garg CC, Harbrianto D, Herrin AN, Huq MN, Ibragimova S, Karan A, Ng CW, Pande BR, Racelis R, Tao S, Tin K, Tisayaticom K, Trisnantoro L, Vasavid C, Zhao Y (2006). Effect of payments for health care on poverty estimates in 11 countries in Asia: an analysis of household survey data. Lancet.

[B13] Peters DH, Yazbeck AS, Sharma RS, Ramana GNV, Pritchett LH, Wagstaff A (2002). Better Health Systems for India's poor.

[B14] Task Force on Health System Research (2004). Informed choices for attaining the Millennium Development Goals: towards an international cooperative agenda for health-systems research. Lancet.

[B15] Ranson M (2002). Reduction of catastrophic health care expenditures by a community-based health insurance scheme in Gujarat, India: current experiences and challenges. Bull World Health Organ.

[B16] Tamil Nadu Census 2001. http://www.census.tn.nic.in/default.htm.

[B17] Devadasan N, Manoharan S, Menon N, Menon S, Thekaekara M, Thekaekara S, AMS team (2004). Accord community health insurance – Increasing access to hospital care. Economic and Political Weekly.

[B18] Garand D (2005). Vimo SEWA India.

[B19] George Alex (2002). Quality of reproductive care in private hospitals in Andhra Pradesh: Women's perception. Economic and Political Weekly.

[B20] van Doorslaer E, O'Donnell O, Rannan-Eliya RP, Somanathan A, Adhikari SR, Akkazieva B, Garg CC, Harbrianto D, Herrin AN, Huq MN, Ibragimova S, Karan A, Lee T, Leung GM, Lu JR, Ng CW, Pande BR, Racelis R, Tao S, Tin K, Tisayaticom K, Trisnantoro L, Visasvid C, Zhao Y (2005). Paying out-of-pocket for health care in Asia: Catastrophic and poverty impact.

[B21] McIntyre D, Thiede M, Dahlgren G, Whitehead M (2006). What are the economic consequences for households of illness and of paying for health care in low- and middle-income country contexts?. Soc Sci Med.

[B22] Sainath P Anatomy of a health disaster. The Hindu 2004 Jul 1.

[B23] Kutzin J (1998). Enhancing the insurance function of health systems: A proposed conceptual framework. Achieving Universal Coverage of Health care.

[B24] Liu X, Mills Anne (1999). Evaluating payment mechanisms: how can we measure unnecessary care?. Health Policy and Planning.

[B25] Bogg L, Hengjin D, Keli W, Wenwei C, Diwan V (1996). The cost of coverage: rural health insurance in China. Health Policy and Planning.

[B26] Brook RH (1994). Appropriateness: the next frontier. BMJ.

[B27] WHO (2000). The World Health Report 2000: Health systems: Improving performance.

[B28] Ranson M, John K (2002). Quality of Hysterectomy Care in Rural Gujarat: The Role of Community-based Health Insurance. Reproductive Health Matters.

[B29] Meessen B, Zhenzhong Z, Van Damme W, Devadasan N, Criel B, Bloom G (2003). Editorial: Iatrogenic poverty. Trop Med Int Health.

[B30] Gertler P, Gruber J (2002). Insuring consumption against illness. The American Economic Review.

[B31] Bonu S, Rani M, Peter D, Jha P, Nguyen SN (2005). Does use of tobacco or alcohol contribute to impoverishment from hospitalization costs in India?. Health Policy and Planning.

[B32] Kawabata K, Xu K, Carrin G (2002). Preventing impoverishment through protection against catastrophic health expenditure. Bull World Health Organ.

[B33] Arhin-Tenkorang D (2001). Health Insurance for the informal sector in Africa: Design features, Risk protection and resource mobilisation.

[B34] Ramesh Bhat (1996). Regulation of the Private Health Sector in India. Int Journal of Health Planning and Management.

